# An assessment of the linkages between GM crop biotechnology and climate change mitigation

**DOI:** 10.1080/21645698.2024.2335701

**Published:** 2024-04-08

**Authors:** Stuart J. Smyth, Peter W. B. Phillips, David Castle

**Affiliations:** a Department of Agricultural and Resource Economics, University of Saskatchewan, Saskatoon, Saskatchewan, Canada; b Johnson Shoyama School of Public Policy, University of Saskatchewan, Saskatoon, Saskatchewan, Canada; c School of Public Administration, University of Victoria, Victoria, BC, Canada

**Keywords:** Carbon sequestration, chemical use changes, GHG emissions, land conservation/use, soil health

## Abstract

This article provides an analysis and evaluation of peer-reviewed evidence on the contribution of crop biotechnology to climate change mitigation and adaption. While there is a range of agricultural technologies and products that contribute to climate change mitigation, this literature landscape analysis focuses on the development of genetically modified traits, their use and adoption in major commodity crops and responsive changes in production techniques. Jointly, these technologies and products are contributing to climate change mitigation, yet the technology, the literature and evidence is still evolving as more sophisticated research methods are used with greater consistency. The literature analysis is undertaken with consideration of the consequential impact that regulatory regimes have on technology development. This assessment utilizes the Maryland Scientific Methods Scale and citation analysis, concluding that GM crops provide benefits that contribute to climate change mitigation.

## 1. Introduction

GM crop technologies are the most rapid instance of innovation diffusion and adoption in the history of agriculture.^1^ Ever since the first commercialization of genetically modified (GM) crops in the mid-1990s, researchers have considered the impact of these new technologies on the economic and ecological footprint of farming. Nearly 25 years of peer-reviewed research of commercialized GM crops in adopting countries establishes that they contribute to yield increases and overall reduce chemical inputs.^2^ The result is that GM crops produce higher global farm incomes and the concomitant changes in land management practices and reductions in chemical use have delivered substantial environmental benefits.^2–4^ As the body of evidence grows, linkages can be established between the adoption of GM crops and their contributions to mitigating climate change. Our objective in this article is to assess how strong those linkages are, and to provide a detailed and structured review of the literature. We fortify this analysis with a standardized evaluation methodology to assess the maturity of the literature and draw conclusions about the strength of evidence linking GM crops to climate change mitigation.

We fully recognize the role of regulatory regimes in creating differential outcomes in GM crop approval, commercial adoption, and the ability of countries to use these technologies as part of their climate change mitigation and adaptation strategy. Differential outcomes arise because of divergent approaches to risk characterization and management, for example in product-based versus process-based regimes or science-based versus precaution-based systems, broadly construed. At issue is the extent to which scientific evidence dominates policy frameworks and drives decision making. Regulatory systems are a function of the scale and scope of the evidence utilized for risk assessment, that is, the submission of increasingly robust risk assessment data should improve the quality and efficiency of the regulatory system, reducing the uncertainty of inconsistent risk assessment decisions, as well as the time required to provide a decision. As of 2019, 4,485 risk assessments of GM crops in 72 countries^a^^a.^The list of 72 countries includes all of the 26 European Union countries as a single country. If counted as separate countries, the number rises to 98. show no elevated risk of GM crop varieties compared with equivalent non-GM varieties.^3^ This number of risk assessments represents the risk assessments required to approve the production or import of a specific GM variety, the actual number of risk assessments from each conducted field trial will be several multitudes higher. Regulatory systems that are science-based have consistently shown to result in consistent and timely risk assessment decisions, compared to those that are precaution-based.^5^ While we firmly believe this evidence should contribute to the design of regulatory regimes and decision-making processes, we are not arguing that specific point here.

Instead, our focus is to assess the literature linking GM crop technologies to sustainability. To foreshadow, we conclude there is evidence across five thematic domains, but the quantity, while not currently substantial, should improve over time. Our analysis provides the evidence for claims about where those improvements can be made. But the regulatory quandary never completely disappears. Countries that approve GM crops may see sustainability dividends but the evidence suggests that countries that do not adopt GM crops will travel down a different road with fewer sustainable agriculture technologies and therefore have fewer tools to manage carbon emissions from domestic crop production. A recent global assessment of GM crop impacts concluded that just one-third of the benefits capable from the technology have been realized the lack of commercialization.^6^ The evidence we have gathered shows that GM crops are capable of being an integral part of both climate mitigation and adaptation. As climates change and governments focus on mitigation and adaptation strategies, innovations such as gene editing are poised to extend and enhance the benefits of GM crops. But the question is, who will benefit?

## 2. Structure of the Literature on the Role of Agricultural Biotechnology Innovation in Climate Change Mitigation

We started with five key research domains to classify the literature: carbon sequestration, chemical use change, greenhouse gas (GHG) change, land use change and soil health.^b^^b.^These domains have been adapted from the FAO’s^91^ five key principles of sustainability for food and agriculture. Based on these searches, we identified a sixth domain, area, that was subsequently included. In 2022, we used those categories to search for relevant impact evaluation literature. Using these five research domains as key words, we searched Google Scholar to identify in excess of 250 candidate articles, that were sorted and assessed against the purpose of presenting evidence of environmental impacts. We found 91 articles that fit our purpose.^90^

These articles were then coded by the five research domains and cross encoded for the key mechanism they were assessing: agronomy, data, genetics and input use, farm structure/size, machinery and policy (Table 1). All of these factors contribute to increased yield and environmental impact. Articles were then assessed based on their citation score to assess their peer reviewed quality of their estimates. The number and average annual cites per year of the articles within each pairings of mechanism and impacts provides one measure of the depth of research across the space, as well as how widely recognized the publications are focused. It is worth noting that there are quite a few mechanisms that have not been tested for their effect on one or more of the environmental impacts.

**Table 1.** Citation impact assessment matrix (# articles/average cites per year as of September 13, 2022).

Assessment Factors	Area	Change in chemical use/toxicity	Carbon sequestration	GHGs	Land use change	Soil health
Agronomy	1/6	15/36	3/9	5/6	2/4	9/6
Data	−	3/47	1/5	−	1/6	2/6
Genetics	−	13/12	1/3	3/13	2/10	−
Farm structure	−	−	−	−	−	−
Machinery	1/15	1/15	−	−	1/45	3/21
Policy	3/7	1/1	−	2/8	−	−

This analysis offers a number of high level insights into the impact of GM crops:

- The carbon sequestration literature establishes that the genetics provided by herbicide tolerant (HT) crops allows for changes in agronomic practices, predominantly the removal of tillage as a form of weed control. Data is limited, but the series of publications by Brookes and Barfoot establish that sufficient data exists at the GM crop adopting country level to estimate the national and global carbon sequestration volumes. Citation of this literature has been minimal, given the more recent publication dates of the published articles.
- There is no evidence to date that changes in farm structure (area) play a significant role. This is not to say that farm structure does not play a role, just that there is no literature to date that quantifies this relationship. With five articles identified, the number of citations is correspondingly low and as above, the more recent article publication dates contribute to lower rates of citation.
- Similarly, while there have been significant changes in seeding equipment over the past 30-40 years that have contributed to the ability of farmers to be better able to direct seed into the stubble of the previous crop, we found no literature is available that quantifies any relationship to increased carbon sequestration.
- Studies show that the genetics of HT and insect resistant crop innovations has resulted in significant agronomic impacts. Citations are concentrated within the domain of chemical use as related to agronomy, while citations relating to genetics are more balanced across the three domains of chemical use, GHGs, and land use.
- In comparisons with the production of commodities prior to the commercialization of GM varieties or in the production of non-GM varieties, evidence is amassing that confirms that GM crops reduce chemical applications. The meta-analysis by Klümper and Qaim^7^ quantifies the benefits of GM crops regarding reduced chemical usage based on the assessment of 147 separate pieces of literature. In many developing countries where small landholders have adopted GM crops, these applications will predominantly be by hand, resulting in reductions of pesticide poisonings. In industrial adopting countries, the applications will be done by mechanized sprayers, with the reduction in chemical use contributing to reductions in GHG emissions. Citations are concentrated within the articles on changes in chemical use, as compared to the other domains. The literature on changes in chemical use has a lengthier publication history than other domains, accounting for this concentration.
- Again, we found there is no literature correlating changes in farm machinery with changes in chemical usage.

The evidence for GHG emissions is most often integrated with carbon sequestration. The evidence concludes that GHG emissions are reduced and carbon sequestration is increased. The leading driver of reduced emissions is the reduction in tillage practices and in-crop chemical applications. Increased carbon sequestration results from the decline in summer-fallow practices and the transition to continuous cropping land management practices.

The category with the most substantial literature is that of changes in land use, which for the purposes of this article is the change from intensive tillage to minimum or zero tillage. Much of the literature discusses the benefits from land use changes, especially increased soil organic matter (SOM) and soil organic carbon (SOC). Increasingly, there is confirmation that the adoption of GM crops is driving changes in land use, especially when it comes to the removal of tillage from crop rotations. The literature that exists is concentrated into agronomic aspects and the collection of data that quantifies the relationship. The only policy-related evidence is an estimate of the impacts that would result if GM crops were to be removed from American farming practices.

Soil health improvements have a limited number of studies, but those that exist report soil health improves following the introduction of GM crops. Part of the literature on soil health relates to improvements in biodiversity, concluding that the impact agriculture has on biodiversity has been reduced following the adoption of GM crops.

## 3. Insights from the Literature on the Role of Agricultural Biotechnology Innovation in Climate Change Mitigation

Innovation plays a key role in multiple aspects of agriculture, from the release of crops with improved biotic and abiotic traits, to seeds matched with equipment that reduces production impacts upon the environment and the collection of data that quantifies changes. This article assesses academic literature on the impacts of agricultural biotechnology on changes in land conservation, carbon sequestration, chemical use and toxicity, GHG emissions, land use and soil health. The discussion below is based on the 91 articles identified by the literature search described above.

### 3.1. Land Conversion

Higher yields and more resilient crops work to lower the pressure on land that otherwise might be left as forest, grasslands, or wetlands. While seldom factored into climate strategies, this is probably one of the most significant contributions an innovation can make to climate change mitigation goals.

Tilman^8^ writing at the beginning of major biotechnological transformations, wrote that doubling of agricultural food production during the previous 35 years using innovations derived from traditional breeding systems was associated with a 6.87-fold increase in nitrogen fertilization, a 3.48-fold increase in phosphorus fertilization, a 1.68-fold increase in the amount of irrigated cropland, but only a 1.1-fold increase in land in cultivation. What he did not consider, however, is that in the absence of more intensive production, more, often marginal, land would have needed to be brought into production, with the attendant loss of carbon sequestration in the soil and water systems. Extending this argument, the Organisation for Economic Cooperation and Development (OECD) has identified 1960 as the turning point when increased production was decoupled from increased land utilization, confirming that from 1960–2020 land use increased 1.1-fold, while food production increased 3.9-fold.^9^

With the advent and widespread adoption of GM seeds, we have a new landscape in which to assess the impact of major technological change. Barrows et al.^10^ examined the impacts of GM crop production on the supply and use of land, finding that GM crops saved 13 million hectares of land from conversion to agriculture in 2010. A similar analysis a few years later shows that if there had been a global ban on GM crops, global cropland would have increased by 3.1 million hectares.^11^ Of this newly needed cropland, 2.5 million hectares would be obtained from the conversion of pastureland, while the remaining 0.6 million hectares would be converted into cropland from global forests, generating a loss in the ecological services from trees. Similarly, Taheripour et al.^12^ estimate that if the US had banned rather than adopted GM crops, a significant amount of land would need to be converted from other crops, cropland pasture, pasture, and forest to meet the global food demand.

Zhang et al.^13^ report that during 1996–2012 there was an increase of more than 370 million tons of food crops, with one-seventh of the increased yield attributed to GM crops in the US. To achieve an increase equal to that delivered by GM crops, they estimate the US would require an addition 300 million acres of conventional crops. These additional 300 million acres would necessarily be lands requiring more fertilizer or irrigation and would be associated with deforestation and other land conversion, both contributing to serious ecological and environmental stress. A report by Brookes and Barfoot^14^ arrives at similar conclusions: for the period 1996–2013 they estimate that biotechnology was responsible for additional global production of 138 million tons of soybeans, 274 million tons of corn, 21.7 million tons of cotton lint and 8 million tons of canola. If those biotechnologies had not been available, maintaining equivalent production levels would have required an increment of 11% of the arable land in the US, or 32% of the cereal area in the EU. Current estimates are that GM crops over the 1996–2020 period delivered 330 million tonnes of soybeans and 595 million tonnes of corn, with all yield increases worth US$261 billion.^15^

These benefits further contribute to the achievement of other land-based environmental goals. For example, Phalan et al.^16^ tested the options of intensive farm management and less intensive “land sharing” options and concluded that birds, for instance, were negatively affected by both practices, but were less harmed by intensive practices.

### 3.2. Carbon Sequestration

As GM crop adoption expanded during the late 1990s and early 2000s, farmers began to experience unrivaled efficiencies in weed control. Prior to herbicide tolerant crops, efficient in-crop weed control options were limited, resulting in farmers predominantly relying on the use of summerfallow for effective weed control. In dryland agricultural production, summerfallow practices resulted in significant soil erosion and loss, as well as reduced moisture conservation. GMHT crops drove the transition from the use of tillage as the lead form of weed control, to continuous, zero tillage land management practices.

These effects have been reported in a series of annual reports on the adoption of biotech crops completed by PG Economics and published in peer-reviewed articles authored by Graham Brookes and Peter Barfoot. The most recent report estimates that 2.4 billion kg of carbon dioxide (CO_2_) was sequestered by GM crop production in 2018.^2^ The authors estimate this was the equivalent of removing over 1.6 million vehicles from the road for one year. The article additionally estimates that the reduction in tillage practices and adoption of zero tillage has resulted in an extra 5.6 billion kg of carbon being sequestered in 2018, which is equivalent to 20.6 billion kg of CO_2_ not being released into the global atmosphere. These savings are equivalent to taking 13.6 million cars off the road for one year. Since 1996, an estimated 302 billion kg of CO_2_ has been sequestered as soil carbon.

Sutherland et al.^17^ surveyed Saskatchewan farmers, exploring how weed control provided by glyphosate allowed farmers to virtually remove tillage from their operations. They found summerfallow decreased from 44% to 1% of hectares between 1991–94 and 2016–19. Carbon accounting results showed that in 1991–94, the average Saskatchewan hectare was a net carbon emitter, releasing 0.03 tonnes/year from tillage. By 2016–19, the average hectare became a net carbon sink, storing 0.12 t/yr from the combination of carbon no longer released from tillage and increased carbon storage from continuous crop production. Soil carbon storage from summerfallow reductions increased from 0.02 t/ha/yr in 1991–94 to 0.42 t/ha/yr in 2016–19. Summerfallow reductions also increase SOC levels by reducing soil emissions from decomposition and by increasing crop residues from continuous cropping.

Applying these values to total Saskatchewan crop production (15.2 million ha) indicates that reductions in tillage between 1991–94 and 2016–19 caused soils to go from being a net carbon emitter of 0.4 million tonnes per year (Mt/yr) to a net carbon sink of 1.9 Mt/yr. From reductions in summerfallow, Saskatchewan carbon storage increased from 0.3 Mt/yr to 6.4 Mt/yr. Canadian agriculture emits about 73 Mt CO_2_ equivalents, or 20 Mt of carbon, each year. Carbon accounting results show that from 2016 to 2019, Saskatchewan soils were annually storing 9–32% of total agricultural emissions through reductions in tillage and summerfallow. Additionally, Saskatchewan soils are currently storing 3–11% of Canada’s required emission reductions of 219 Mt CO_2_ equivalents in the Paris Accord each year.

There is an abundance of literature on changes in land use, reductions in GHG emissions and increases in carbon sequestration, but little of this literature frames the changes specifically as resulting from the adoption of GM crops. Awada et al.^18^ for example, built a model to account for different farming practices (i.e., conventional, minimum and zero tillage, summerfallow, crop rotations and residue retention) and input usage rates (i.e., fertilizer and fuel) to estimate how they affect GHG emissions in different soil climate zones and provinces in the Canadian prairies region. The adoption of sustainable practices led to an 80% decline in GHG emissions in the crop sector between 1985 and 2016. While GM crops were not explicitly explored, they were noted as critical factor in opportunities to use advanced soil conservation methods and in reducing fertilizer and fuel usage.

Most scholars agree that increased carbon sequestration and the benefits of this are inextricably linked to the adoption of GM crops and the resulting changes in tillage and land management practices. Yet very few articles report specifically on the role of GM crops. Consequently, it is not possible to estimate what portion of the measured benefits of improved farm practices are strictly due to GM crops. Kern et al.^19^ undertook research quantifying the net balance of emitted and assimilated CO_2_ due to the application of crop protection treatments on farms. The final CO_2_ balance is positive and may reach multiples of up to a factor of nearly 2000. Their findings indicate that crop protection products in particular contribute to GHG emission reductions and mitigation in agriculture.

### 3.3. Changes in Chemical Use/toxicity

One of the major purposes of GM seeds is to pair crop genetics with less toxic chemicals (in the case of weed management), to reduce costly supplemental chemical applications (in the case of Bt traits) or to manage viral disease that might damage yields. Depending on the crop and the purpose, GM fields either use different chemicals, fewer chemicals or no chemicals. In a few developing countries some pairings have generated a greater use of chemicals, but mostly because weed or pest pressure was now manageable in a cost-effective way that did not previously exist.

One crop that has been extensively studied is canola. There is a general consensus that the amount of herbicide active ingredient per hectare for GM canola production in Canada has decreased, that herbicides are applied at lower rates, have lower environmental impact (EI) and that producer exposure has been reduced. The Canola Council of Canada^20^ examined practices and impacts in 1999–2000, when approximately three-quarters of the canola acres were GMHT varieties. The study estimated that the lower herbicide-use on GMHT canola fields in Western Canada was the equivalent of 6,000 fewer tonnes of herbicide application in 2000, a reduction of 40%.

Brimner et al.^21^ examined the changes in herbicide use due to GMHT canola adoption between 1995 and 2000, finding that herbicide use on conventional canola increased by 30% while herbicide use on GMHT canola decreased by 20%. They conclude that the EI for GMHT canola dropped 37%, but rose 56% for conventional canola. The authors noted that their study assumed GMHT canola was only sprayed with the corresponding herbicide and not tank-mixed with other herbicides, which could lead to under-estimating the actual application rate; conversely, they ignored any herbicides applied to conventional canola fields as a burn-off prior to seeding. Kleter et al.^22^ compared conventional and transgenic canola crops in the US over four years, finding that the application of herbicide active ingredient was 30% lower in GMHT canola than in conventional canola crops. The total EI per hectare was 42% lower, the ecological impact was 39% lower and the farmer impact was 54% lower. Brookes and Barfoot^23^ compared and aggregated environmental impact quotients (EIQ) values for GM and conventional crops in Canada and the US, concluding that between 1996 and 2008 the amount of active ingredient applied to canola decreased by 13.7 million kg or 18%, with a corresponding drop of 24% in EI. The study assumed that farmers applied herbicide at the recommended maximum level, which created the potential for over-estimation of application and underestimating the decline in usage and the net overall benefit. Leeson et al.^24^ examined trends in herbicide use in canola production, comparing weed surveys from the three prairie provinces from 1995 to 1997 against similar surveys from 2001 to 2003, concluding that herbicide use dropped 12% and the EI fell 22% per hectare.

Significant changes in use and application of herbicides have occurred in canola weed management practices in Western Canada.^25^ Comparing canola production in 1995 and 2006, the toxicity of herbicides applied to canola decreased by 53%, producer chemical exposure decreased by 55% and 1.3 million kg of chemical active ingredient that would have been applied with non-GM seeds was not applied. The cumulative environmental impact per hectare (EI/ha) of the top five herbicides applied in 1995 was 46,715, while the figure for the top five herbicides applied in 2006 was 29,458. If GMHT canola had not been developed and Canadian canola farmers continued to use previous production technologies, the amount of active ingredient applied to control weeds in 2007 would have been 38% above what was actually applied. Brookes and Barfoot^2^ updated the canola situation in Canada, estimating a reduction in chemical active ingredient of 34.3 million kg, representing a 35% reduction in the environmental impact.

Other crops have had similar impacts. In their most recent assessment of the environmental impacts from GM crops, Brookes and Barfoot^2^ identify that the production of GM soybeans in Canada from 1997 to 2018 resulted in the reduced application of 4.56 million kg of active chemical ingredient, leading to a 23% reduction in the EI of chemicals applied for soybean production. Applying the same assessment to soybean production in Brazil over the 1997–2018 period identified an increase of 24 million kg of chemical active ingredient which, when offset by the lower impact factor of the chemicals applied, delivered a 7.2% decline in the environmental impact. Extended to global soybean production, the authors identify an increase of 5 million kg of chemical active ingredient, an increase of 0.1%, but a corresponding reduction in the environmental impact of 12.9%. An important caveat to the change in global chemical active ingredient is that Brookes and Barfoot do not account for changes in the production of soybeans during the period, as soybean production in the US alone has increased by 20 million acres. Additional acres of soybean production will account for increased chemical application, which may mask lower rates of application. Global soybean production has significantly increased since 1995, rising from 25 million metric tonnes (MMT) to 240 MMT. The increased production of soybeans in Latin America between 1990 and 2016 has been staggering, with Argentina increasing by 286%, Brazil by 248%, Paraguay by 281% and Uruguay by 3,474%.

To better assess the nature and impacts of changes in pesticide use, an evaluation of chemical use by US corn and soybean farmers from 1998 to 2011 was conducted.^26^ On average, adopters of GM glyphosate tolerant (GT) soybeans used 28% (0.30 kg/ha) more herbicide than non-adopters, adopters of GT corn used 1.2% (0.03 kg/ha) less herbicide than non-adopters and adopters of GM insect resistant corn used 11.2% (0.013 kg/ha) less insecticide than non-adopters. When pesticides are weighted by the EIQ, however, it was identified that (relative to non-adopters) GM adopters used about the same amount of soybean herbicides, 9.8% less corn herbicides, and 10.4% less corn insecticides.

An assessment of atrazine use for corn production in Wisconsin examined what impact atrazine use restrictions had on the range of weed management practices.^27^ A survey of farmers was done to gather data from both areas where atrazine restrictions had been implemented and areas that had no restrictions. The results found that restricting the use of atrazine increased the adoption of herbicide tolerant (HT) corn varieties, which then contributed to an increase of conservation tillage practices.^c^^c.^The combination of atrazine restrictions and increased HT corn production contributed to a reduction in herbicide modes of action that were being applied. Dong et al.^27^ concluded that the reduction in the diversity of weed control options may contribute to an increase in the potential for herbicide resistance in weeds. The authors highlight that the regulatory efforts to restrict atrazine in groundwater might have a knock-on effect of more herbicide resistant weeds, which may need to be controlled by tillage, which in turn works to increase soil erosion and a deterioration in water quality.

The commercialization of Bt cotton resulted in substantial reductions in chemical application. Between 1996 and 2018, Brookes and Barfoot^2^ identify a global reduction of 40 million kg of chemical active ingredient for HT cotton production, reducing the environmental impact of the chemical applied by 12.2%. Insect resistant cotton has resulted in a reduction of 331 million kg of active ingredient, a 34% reduction in environmental impact.

The 2002 commercialization of Bt cotton in India, with its millions of small landholders, provided an opportunity to assess GM crop adoption for developing world farmers. Qaim^28^ assessed Bt cotton adoption in India (based on 2001 field trials), noting that prior to commercialization, farmers were losing an estimated 50–60% of potential yield to insect pests. The analysis found that GM seeds bumped yields by an average of 58% higher and cut pesticide costs by 50%. Subramanian and Qaim^29^ report that after four years of production, Bt cotton yields were 37% higher and pesticide use dropped by 41%. Additional socio-economic benefits were also measured, with the most noticeable impact being increased use of paid female labor. Subramanian and Qaim estimated that Bt cotton-adopting households increased their incomes by 82% and households that were defined by the FAO as vulnerable (i.e., income of <US$2/day) increased their incomes by 134%.

Further research by Qaim^7^ shows that the application of cotton pesticides has fallen by between 0.95 and 1.3 kg/acre of active ingredient. This results in a cost savings of 879–1,284 rupees (US$13–19) per acre. In India pesticides are applied by farmers walking through the field using a backpack sprayer, in most cases with little to no protective clothing. Millions of cases of acute pesticide poisonings were reported every year. The adoption of Bt cotton reduced the number of cases of pesticide poisoning, estimated to range between 2.4 million and 9 million annually. This annually saves the Indian Ministry of Health an estimated US$14–51 million.^30^ Not only has the environment and farmer health benefited, but so too has yield and profitability of Bt cotton adopters. While adopters pay a higher price for seed, the cost is more than offset by the 24% increase in yield when compared to non-Bt cotton. Profits rose even more dramatically, by an estimated 1,877 rupees (US$28) per acre or 50%. In 2012, it was estimated that 27 million acres were planted to Bt cotton, representing 95% of cotton production in India, generating a net gain for farmers of US$1 billion. Cotton production has increased in India to such an extent that it is now the second largest producer, trailing only China.

China has invested heavily in biotechnology and has been a strong adopter of Bt cotton. Based on a 1999 survey of cotton farmers in northern China, Pray and Huang^31^ provided the first insights into the impacts of Bt cotton. Their research measured economic, income distribution, environmental and health effects. While not easy to quantify due to farmer-to-farmer sales and seed saving from year-to-year, the authors estimate that early adoption ranged from 8% to 27%. The most substantial impacts from Bt cotton adoption was the environmental and health benefits resulting from reduced pesticide applications. The adoption of Bt cotton allowed farmers to spray less frequently, in some instances dropping from 30 applications per season to three, but more commonly from 12 to 3–4.

Huang et al.^32^ updated their results on Chinese Bt cotton following a decade of commercial production. They documented a drop in bollworm infestations, not only in Bt cotton fields, but in all cotton fields in parts of China. In some non-Bt cotton fields the amount of insecticide used dropped from in excess of 40 kg/ha to less than 10 kg/ha. Across the entire sample region, insecticide applications dropped from 14 kg/ha to 4 kg/ha.

Similar reductions have been observed in the production of GMHT corn, with a reduction of 242 million kg of chemical active ingredient, a 12.1% reduction in the environmental impact.^2^ GM Bt corn has reduced chemical active ingredient application by 112 million kg, a 63% reduction in the environmental impact.

Other crops are only sparsely studied. The commercialization of Bt brinjal in Bangladesh has resulted in both a reduction in the amount of chemical applied in production, as well as a reduction in the EI of the chemicals applied. Ahmed et al.^33^ found Bt brinjal in Bangladesh has increased yield by 20%, decreased pesticide costs by 38%, and cut the toxicity of pesticides applied by 76%. Macall et al.^34^ found that 84% of farmers growing Bt corn in Honduras applied no pesticides, while realizing yields 50% higher than non-BT crops.

In a wider assessment of literature that included journal articles, government reports as well as industry and organization reports, Klümper and Qaim^7^ delivered a meta-analysis of 147 studies that showed that chemical use declined by 37%, yields increased by 22% and farmer profits increased by 68%.

One study has been conducted that examines the potential economic and environmental impacts that would arise if restrictions on glyphosate use resulted in the world no longer planting GMHT crops.^35^ This would generate an annual loss of global farm income of US$6.76 billion and drops in global soybean, corn and canola production, equal to 18.6 million tonnes, 3.1 million tonnes and 1.44 million tonnes, respectively. There would be an annual environmental loss associated with a net increase in the use of herbicides of 8.2 million kg of herbicide active ingredient (+1.7%), and a 12.4% net increase in the environmental impact, as measured by the EIQ indicator. Also, there would be additional carbon emissions arising from increased fuel usage and decreased soil carbon sequestration, equal to the equivalent of adding 11.77 million cars to the roads.

A final, indirectly-related aspect of reduced chemical use is further innovation in mechanical options. Walsh et al.^36^ assess one such innovation, the Harrington Seed Destructor, which destroys all seeds that pass through a combine, such as unharvested crop seeds and weed seeds. Their study reported that using this innovation reduced viable weed seeds by more than 95% when harvesting wheat, barley and lupines. The dispersion of weed seeds at harvest using normal combines and harvesters results in the need for herbicide applications the following spring. Using the seed destructor led to substantial reductions of weed seeds at harvest, which created the opportunity for reduced herbicide applications in the subsequent crop years. The adoption of this technology would further contribute to reducing chemicals applied to control weeds.

### 3.4. Changes in Greenhouse Gas Emissions

**Figure 1.**
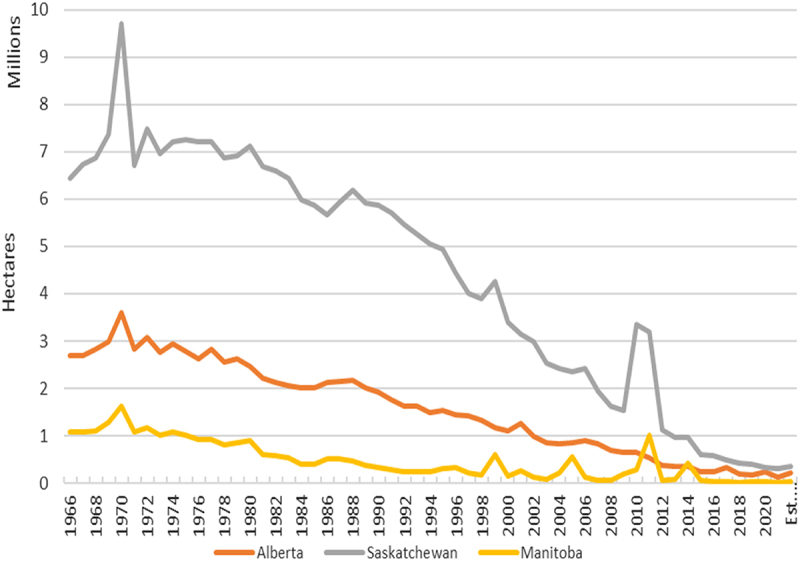
Canadian prairie summerfallow area, 1966–2022.

Source: Statistics Canada^37^

The improved ability to control weeds has resulted in farmers transitioning their land from having summerfallow as a core part of their crop rotations, to near full removal of summerfallow practices. Figure 1 illustrates just how significant this reduction has been across the Canadian prairie provinces of Alberta, Saskatchewan and Manitoba. In 1995, the first year GM canola was produced there was 6.8 million ha of summerfallow across the three provinces. By 2022, this dropped to 613,000 ha, a decrease of 91%.

**Figure 2.**
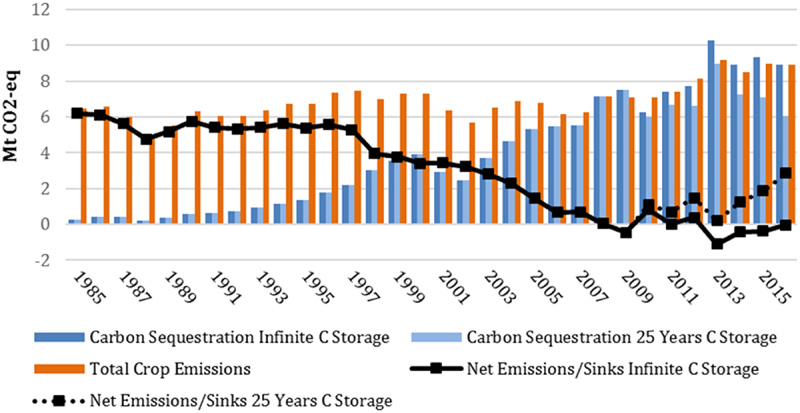
Net GHG emissions/carbon sink in Saskatchewan’s crop sector, 1985–2016.

**Source**: Smyth and Awada^38^

The reduction in summerfallow increases the area of crop production, which changes GHG emissions. Depending on the region of crop production, fewer field passes would be made by machinery when land is producing crops, compared with summerfallow. In other regions, there may be little difference. One estimate of GHG emissions in Saskatchewan,^38^ indicates that since 2008, crop production has been a net carbon sink (Figure 2). Between 2013 and 2016, emissions remained relatively constant, with the continuous, zero tillage cropping resulting in increased carbon sequestration, hence the net GHG sink.

Economic studies which model the effects of conservation tillage adoption on soil properties have also shown positive impacts on carbon sequestration. A study by Grant et al.^39^ investigated how changes in management practices affect GHG emissions, finding that the average net reduction in emissions from converting to zero tillage was 0.61 Mg CO_2_ equivalents per ha, per year in Canada. As noted above, Awada et al.^18^ found the sustainable practices that combine better tillage and new genetics led to an 80% decline in GHG emissions in the Canadian prairie crop sector between 1985 and 2016. After 2005, emissions dropped 53%, more than is required to meet the 2030 Paris Accord target. In Alberta, crop production was a net GHG sink between 2013 and 2016 and between 2006 and 2016 in Saskatchewan.

Shrestha et al.^40^ conducted a GHG inventory analysis of Canadian canola production between 1986 and 2006, finding that reductions in summerfallow sequestered 0.4 Mg CO_2_ equivalents per ha, per year (ha/yr), while conservation tillage adoption sequestered 0.2 Mg CO_2_ equivalents/ha/yr. MacWilliam et al.^41^ found that GHG emissions from one tonne of canola production decreased across all Canadian prairie soil zones from land use and land management changes between 1990 and 2010.

The delayed adoption of GM canola production in Australia was studied by Biden et al.,^42^ finding that the delay cumulatively resulted in the application of an additional 6.5 million kg of chemical active ingredient. The application of these additional chemicals were done through an additional 7 million field passes, requiring 8.7 million liters of diesel. The environmental impact of the additional chemicals applied was 14% higher than would have been the case if GM canola had not been subjected to an adoption moratorium. Finally, an estimated 24 million additional kilograms of GHGs were released due to the non-adoption of GM canola.

An assessment of EU agricultural GHG emissions concluded that had the EU adopted GM crops in a timely fashion as North America, total emissions would be reduced by 7.5% of the EU’s total agricultural GHG emission.^43^ This amounts to 33 million tonnes of CO_2_ annually.

### 3.5. Land Use Change

Herbicide tolerance allows farmers to control a broad spectrum of weeds through in-crop applications without damaging crops. Farmers who grow HT canola are more likely to adopt conservation tillage practices.^44,45^ Similar results have been seen in HT soybean production. In 1997, soon after the introduction of HT soybeans, twice the number of acres were planted under no tillage with HT soybean than those with conventional soybean in the US.^46^ Results from a 2006 survey of 1,195 US farmers across six states (Iowa, Illinois, Indiana, Mississippi, North Carolina, and Nebraska) found a complementary relationship between the adoption of conservation tillage and HT crops. Of farmers in the survey who had previously used conventional tillage, 56% adopted minimum or no tillage systems following the introduction of HT crops and 25% of farmers who had been practicing minimum tillage shifted to no tillage.^47^ Similar results from a 2009 survey of US farmers showed 80% of respondents believed there was less tillage in HT production than in conventional production.^48^ The complementary relationship between these technologies has also been studied using economic and econometric modeling techniques. Numerous studies have concluded in favor of this relationship (e.g.,^49–51^)

To obtain adequate weed control in summerfallow, a minimum of 3–4 annual tillage operations were required in Western Canada^52^ and often up to eight passes were made, depending on the region.^53^ Leaving a field fallow also results in continued microbial activity and decomposition of available residue in the soil but lacks any residue input, an important factor in increasing soil organic carbon (SOC) stocks, leading to a decrease in SOM.^54,55^ The combined effect of the frequent tillage and lack of crop residue leads to increased soil erosion and in many cases, an unintended decrease in soil moisture. Consequently, SOC stocks typically decrease during fallow years.^56^ Therefore, decreasing summerfallow area contributes to increased SOC levels by reducing soil emissions and, through the shift to continuous cropping, increasing crop residue levels.

Studies looking at the impacts of reducing summerfallow have been conducted using modeling techniques. For example, Grant et al.^39^ modeled the impact of changes in management practices on Canadian emissions between 1979 and 2029. They predicted that the net emission reduction from the elimination of summerfallow would be 0.56 Mg CO_2_ per ha, per year. In a study of the long-term farm management effects on SOC, Sperow^57^ used 2006 International Panel on Climate Change estimates for SOC factors to study the effects of reducing summerfallow. His results showed that the effects of eliminating summerfallow were relatively modest, increasing SOC stocks by 0.16–0.24 Mg C per ha, per year and contributing about 3% of total potential sequestration from all activities studied. More recently, Rosenzweig and Schipanski^58^ used satellite data to study cropping patterns in Colorado, Kansas, and Nebraska, finding a decrease in summerfallow use from 48% to 33% of dryland cropland. The authors assessed the impacts of this cropping intensification on carbon sequestration, concluding that sequestration increased by 38% due to the adoption of mid-intensity and continuous rotations in place of summerfallow.

Continuous cropping increases crop residue levels, which contributes to increased accumulation of SOC.^59^ Crop residues include any roots, stems or other plant material left in the field after harvest. The amount of crop residue is affected by crop yield and biomass. Early in the 20th century, crop residues were considered unfavorable and farmers correspondingly took steps to remove residues from their fields. Often, residues were burned or used as livestock feed and bedding. However, by 1980 the value of carbon sequestration and the beneficial contribution made by crop residues to reducing net GHG emissions began to be recognized.

Although many past studies assumed that the rate of carbon input to the soil is similar for all crop types, more recent studies have shown that above- and below-ground crop biomass varies significantly between crop types. Carbon-to-nitrogen ratios impact changes in SOM and SOC levels as well. For example, soybeans have a relatively low carbon-to-nitrogen ratio and correspondingly, soybean crops typically result in lower carbon inputs to the soil.^60^ Therefore, crop type is an important factor to consider when estimating changes in SOC. Gan et al.^61^ calculated carbon allocation coefficients for various crops which represent how much carbon is returned to the soil from each part of the plant relative to total carbon mass. They found, on average, that pulses had the greatest allocation coefficient for seed production and conversely, oilseeds had the greatest coefficient for straw. For all crops, the allocation coefficients for the roots were lower than for the grain or straw.

The reduction or elimination of disturbance to the soil layers in a minimum tillage or no tillage (NT) system also benefits farmers economically by reducing soil erosion, which has substantial effects on agronomic performance. Bakker et al.^62^ estimated that in mechanized agriculture, for every 0.1 m of soil loss, crop yields are reduced by 4% in the EU and North America. No till systems leave the majority of crop residues on the soil surface instead of incorporating them into the soil profile, which help to increase SOM content and decrease the negative impacts of erosion. Additionally, crop residues on the soil surface will reflect sunlight and conserve moisture by lowering the temperature of the soil and protecting it from high evaporation levels.^63,64^ All of these impacts have an effect on soil quality which affects agronomic performance and crop yield.

Carpenter^65^ examined the biodiversity impacts resulting from GM crop adoption, concluding that GM crops reduce the impact that agriculture has on biodiversity. The article reviews significant literature on the impacts of GM crops on crop diversity, non-target soil organisms, weeds, land use, non-target above-ground organisms and area-wide pest suppression. The review includes evidence from studies conducted in China, Denmark, Germany, Portugal, Switzerland and the USA.

### 3.6. Changes in Soil Health

An important element of reducing net agricultural GHG emissions is improving levels of soil carbon sequestration. Carbon sequestration offsets positive emissions by transferring carbon from the atmosphere into secure soil storage pools through the process of photosynthesis. The CO_2_ that is removed from the atmosphere by plants and transferred into the soil becomes soil organic carbon; thus, increases in SOC represent increased carbon sequestration. Each tonne of carbon in the soil represents about 3.67 tonnes of CO_2_ sequestered in the past.^66^ Capacity of the soil carbon storage pools are estimated to be four times the vegetation carbon pool and three times the atmospheric pool,^67^ with the capacity of each pool depending on soil characteristics, precipitation and climate.^68^ The capacity of these storage pools is large, but previous studies have indicated they are finite,^69^ with several previous studies estimating maximum storage pool capacities being reached 15–20 years after adoption of new management practices.^70,71^ However, small changes in sequestration rates can cause substantial changes in carbon equilibrium timeframes^72,73^). More recent studies suggest that through careful management, strategies may be developed to increase the sequestration potential of storage pools.^74^ Paustian^75^ identifies three management practices that contribute to increased levels of SOC: 1) minimizing soil disturbance and erosion; 2) maximizing crop residue levels; and 3) maximizing efficiency of water and fertilizer use. All three of these correlate to the adoption of GM crops, as decreasing the frequency of tillage operations and increasing cropping intensity by reducing summerfallow are strategies which help to achieve these goals.

Numerous soil science studies have examined the effects of conservation tillage adoption on carbon sequestration. While these studies do not necessarily identify the contributions of GM crops, GM crops have resulted in reduced tillage, therefore the results of increased soil conservation practices are applicable to the continuous cropping, zero tillage production of GM crops. In 2002, West and Post conducted a survey of the extensive soil science literature to quantify carbon sequestration rates, finding an average increase of 0.57 ± 0.14 Mg carbon per ha, per year from conservation tillage adoption. McConkey et al.^76^ found SOC increases ranging from 0.067 to 0.512 Mg per ha, per year across Saskatchewan, with variations resulting from soil type and location. Liebig et al.^77^ studied emission mitigation strategies specifically in the Northwestern US and Canada, concluding that although the effects of crop management on SOC varied, no till systems in continuous, dryland cropping resulted in an average SOC increase of 0.27 ± 0.19 Mg per ha, per year. More recently, Aziz et al.^78^ studied the impact of tillage practices on soil quality, which was defined based on an index made up of a range of biological, chemical, and physical soil properties. Results of their study found that no till methods resulted in 30% higher soil carbon than conventional tillage. Similarly, Nath and Rattan,^74^ studied differences in soil aggregation and SOC resulting from changes in tillage practices. Results of their study showed that corn managed under a no till system sequestered 35–46% more carbon than conventionally tilled corn.

The positive effects of converting to NT systems may vary based on the time period and soil depths used for analysis. A meta-analysis by Angers and Eriksen-Hamel^79^ suggests that, in the short-term, NT systems may not have a net positive contribution to SOC stocks due to accumulation of carbon at the soil surface. However, their results show that the benefits of a NT system likely increase with time (>10–15 years). Similar results from Blanco-Canqui and Lal^80^ indicate that gains to SOC as a result of decreased tillage are restricted to the surface soil layers. VandenBygaart et al.^81^ reported SOC increases in both the 0–15 and 15–30 cm depths in Western Canadian soils from the adoption of NT, yet improvements were higher in the 0–15 cm depth. Though conservation tillage systems might re-distribute residual carbon throughout the soil profile better than NT in the short-term, the net carbon gain resulting from a NT system in the long-term offsets this redistribution of carbon to deeper soil levels.^82^

A literature review and meta-analysis by Lee et al.^83^ indicated profitability increased for farmers using GM crops and conservation tillage. The combined adoption of both technologies reduced agricultural impacts on the environment and often improved soil and water quality. Soil quality improvements have been associated with reduced tillage, decreased erosion and increased carbon sequestration, whereas water quality improvements are associated with greater post-emergent herbicide use that limits soil exposure and subsequent runoff.

The lack of proper soil nutrients is commonly the main factor limiting yields, particularly nitrogen and phosphorus. The development of plant growth-promoting bacteria has been found to increase plant growth and plant biomass, reduce plant leaf water loss, enhance root development and increase photosynthetic efficiency.^84^ These crop enhancements were identified in the production of soybeans, corn, rice, tomatoes, peppers, sunflowers, canola, cotton, peanuts, oats, sugar cane and chickpeas. The ability to mitigate plant stresses through improved nutrient uptake would result in a reduced reliance on synthetic fertilizers to provide adequate crop nutrients, helping to mitigate climate changes. To date, applications of these technological innovations that increase nutrient use efficiency are limited to laboratory experiments and small plot evaluations and have not been approved for commercialization. Large scale adoption assessment would be needed to better quantify the impacts.

## 4. Methodology and Analysis

While citations tell us something about the peer reviewed opinion of the quality of the estimates, they are only partial indicators of quality. High citation can be both a signal of good and bad method while much of the evidence vital to decision making is often lightly cited. There is increasing interest in other measures of quality of evaluations. The Maryland Scientific Methods Scale was developed in the 1970s to communicate to scholars, policymakers and practitioners in the simplest possible way the methodological quality of various evaluative studies, by coding them by their methodological quality. Using the approach developed by Radcliffe,^85–89^ which offers a fine-grained hierarchy for evidence, ranging from ad hoc confirmatory efforts (level zero) through to well-structured systematic reviews, which at their apex involve meta analyses of a set of repeated randomized controlled experiments. This second phase of our analysis complements the robustness of the above literature review. This analysis quantifies which literature has theory, methods and evidence, three essential elements of strong evaluation. Quantifying the size of the evidence pool, allows for differentiation between where there are a few, disconnected studies showing benefits versus where there is a defined body of evidence firmly supporting benefits. The MSM Scale offers seven levels of evidence including:

- Level 0: Those reviews that are confirmatory and have limited value by themselves. Anecdotes, case studies and general reports that are designed to justify effort often have little probative value if they are the only evidence.
- Level 1: Two types of review fit here: cross-sectional ex post comparison of treated groups with untreated groups or a before-and-after comparison of a treated group, without any comparison with an untreated group or any use of control variables. These types of analysis can identify correlations but often are not able to assign causality, partly because without any formally controlled counterfactual, there is no way to definitively show that the measure caused any observed results.
- Level 2: Studies at this level use control variables to do either cross sectional or before and after comparisons, albeit still without any untreated comparison groups. These types of studies may be able to establish a causal order but fail to rule out many threats to internal validity, in that there may be other explanations for what was observed.
- Level 3: The minimum standard for substantive evaluation is that the measure be structured as an experiment, with a well-defined source population that fits with the policy area, and where there are both treated and non-treated subjects that can be assessed for behavioral responses. In effect these could be considered a quasi-experiment or randomized control trial (RTCs). Both causality and scale and scope of impact of the measure can be discerned in such studies.
- Level 4: This level of evidence draws on the repeated use of RCTs to control for other variables. This helps to remove the chance of spurious correlations from a single assessment of any venture.
- Level 5: Level 3 and 4 evidence is drawn from purposeful construction of in-experiment subjects and controls. Level 5 studies remove that restriction, applying the same experimental methods to randomly assigned populations, so that the causal assumptions can be validated more generally.
- Level 5*: Once a body of evidence through a range of Level 3 and 4 studies has accumulated, sometimes there is value in doing a study of studies, to discern the meta results and sensitivity analysis of the influence of different modeling approaches and assumptions on the derived outcomes.

We used this rubric to code all the literature, undertaking the following steps:

- In order to have a proper ordering, we rebased this Maryland Scientific Methods (MSM) scale to 0 = 1, 1 = 2 and so on to 5* = 7.
- We first coded every article by its impact: area, chemicals, carbon sequestration, GHG, land use and soil health.
- We entered in their citation rate as of September 13, 2022.
- We coded every article for where it would fit in the MSM scale, from 1-7.
- We then ran reports to understand the nature and quality of the work being done.

Our assessment illustrates where this literature is still emerging (Table 2). We found 74 articles that offered structured evidence of environmental impacts. In a few cases, the articles looked at more than one mechanism (agronomy, data, genetics, farm structure, machinery, or policy) and a few explored more than one impact (area, carbon sequestration, chemicals, GHG, land use, soil health). Those articles that are multifactorial are included more than once.

**Table 2.** Overview of the methods of evaluation.

MSM coding	Count	Average citations	Average age of literature (yrs)
1	4	42.5	16.5
2	25	103.4	10.9
3	21	129.3	9.1
4	10	163.5	11.2
5	5	77	7.0
6	0	na	na
7	9	547.5	11.8

One can see that some of the oldest work was really case studies and anecdotal evidence (MSM 1) and that that work has a relatively low citation rate. Investigators have taken on more advanced methods (MSM 2–4) as the technology has matured and that body of work is getting taken up and cited. More advanced RCTs with counterfactuals (MSM 5) is just getting going. We found only five articles that did level five RCTs and they were on average only seven years old but were relatively lightly cited. We could not find any that have run these studies long enough to qualify for MSM 6, which requires repeated RCTs to remove sampling bias. We found a range of meta analyses, but one must keep in mind these surveys can only be as good as the underlying literature, which still lacks full rigorous RCTs.

Different impacts have been differentially studied. So far chemical, soil health and GHG effects are well articulated, albeit without repeated, full randomized control trials with confounding variables (which would code MSM = 6). Nevertheless, we found more than 10 articles in each area, and at least one meta-study in each domain (Table 3). There were fewer articles about carbon sequestration but we found they used more a somewhat more rigorous methodology and offered at least one meta-study (MSM = 7). Relatively speaking the literature on area and land use is a bit weaker in terms of method, without any full randomized controls or meta studies. On average, the literature is 11 years old, with the oldest dating back to 2000. Apart from the impact on area, most of the literature is distributed around the mean of 11 years.

**Table 3.** Citation analysis.

	Area	Chemicals	Carbon sequestration	GHG	Land use change	Soil health
Number of articles found	5	32	7	12	7	13
Average age (years)	8	11	11	10	12	11
**MSM scale**						
Lowest	2	1	2	2	1	1
Highest	4	7	7	7	4	7
Average rating	2.8	3.2	3.6	3.8	2.7	3.6
**Citations**						
Minimum		0	3	0	24	11
Maximum		7,430	2,582	281	626	452
Average	79	369	454	65	163	104
Average/year	8	26	25	6	13	10
Average to average/year	10	14	18	10	13	11
Average cites/year per MSM point	2.9	8.0	7.0	1.7	2.9	2.9

Source: Author’s calculations.

Comparing citations tells us something about the maturity of the literature. All other things being equal, more highly cited articles are generally recognized as providing more probative value. Successive comparisons offer some insights. There is both absolutely more literature on the chemical impacts: the total citations are higher, the average cites per year (adjusting for the age of the literature) is almost at the top and the average cites per year per MSM point is strong.

Carbon sequestration, GHG, land use and soil health all have some strengths, but the literature is a bit thin, which suggests one should be cautious about making judgments on its messages. By definition repetition is necessary to confirm the impacts.

## 5. Discussion

A recent assessment of the global impacts from GM crops found that in the absence of GM crops, an additional 3.4% more land would have been required to produce the equivalent yields.^6^ This additional land would have required the conversion of unsustainable marginal land into crop production as well as cutting down additional forested lands. The study further concluded that regulatory bans on the commercialization and adoption of GM crops restricted the benefits resulting from GM crop production to be just one-third of their full potential. The limited adoption of GM crop technology clearly highlights the quandary that food security, sustainability and climate change mitigation all face. So far, all precaution-based regulatory systems have rejected or seriously impeded the full commercialization of GM crops, contributing to greater food insecurity, the continuation of unsustainable agricultural practices, higher GHG emission levels and reduced carbon sequestration.

The literature identified for this article, highlights two patterns of interest. There are two predominant concentrations, the first is that produced by Brookes and Barfoot with the second from organizations, institutions and universities located in the Canadian provinces of Alberta, Manitoba and Saskatchewan. The integration of the research across more than one domain is evidenced by citations in Sections 3.1–3.6. Additionally, the cross citations illustrate the research complexity of research regarding climate change mitigation. The cross citation of the key literature illustrates that successful efforts at mitigating climate change require a systems approach, that is, innovative crop breeding resulting in herbicide tolerant crops that provide ongoing, effective weed control, allowing for the continuous removal of tillage, which facilitates GHG reductions and increased carbon sequestration and improved soil health.

While the challenges facing agriculture are numerous, two key ones stand out: mitigating climate changes and contributing to the United Nation’s (UN) Sustainable Development Goals (SDGs), especially the first three of reduced poverty, less hunger and better health. Innovation will be the cornerstone of ensuring that agriculture and food product is able to be done such that yields and nutrition are maintained through climactic change. Discussions at the Convention on Biological Diversity’s Conference of Parties Meeting on Biodiversity (COP-15) held in Montreal in December 2022, highlighted the visceral opposition of some to protecting biodiversity by such beneficial and safe technologies as the use of genetic modification technologies. Rejecting empirical evidence of the benefits from GM crops ensures that these contributions to mitigating climate change and achieving the SDGs will be dramatically minimized in the future.

Efficient, repeatable, and timely regulation is crucial for successful innovation. Evidence is vital for advancing effective regulation. The commercialization of GM crop varieties over the past 30 years confirms that product-focused regulatory frameworks grounded in empirical evidence provide the efficiency, repeatability, and timeliness required to further incentive R&D investments. A lack of trialing and evaluation in other markets impairs full adoption. R&D investment into variety development in the EU has declined as a result and more resources have flowed to countries with product-based regulatory systems, such as Argentina, Australia, Brazil, Canada, and the USA. The lack of local evidence impairs innovative crop variety development, undermining the goal of mitigating climate change and achieving the SDGs.

## 6. Conclusions

A growing volume of research contributes to quantifying the environmental benefits from GM crops, which helps regulators, scientists, and risk assessment experts make decisions that advance plant technologies that contribute to mitigating climate change and achieving the SDGs. In the absence of these innovative technologies, countries will find it increasingly challenging to meet both their climate mitigation and SDGs objectives. Scientifically indefensible regulations are a large obstacle to safe technological solutions to many of the myriad problems and challenges facing agriculture and climate change mitigation. Better and more evidence will be necessary to move forward.

The available literature offers some compelling evidence that GM crops contribute substantially to climate mitigation, but more is needed to fully understand the complex interactions between variable cropping systems, the local ecosystem and global climate and the local economic and social systems. Regulators and policy makers have cited some of the results of the impact studies explored above in support of regulatory and policy decisions, but mostly without explicit attention to the quality of their underlying design or the complex interaction of the various subsystems. Complicating this is both the paucity of randomly controlled trials and the lack of studies that quantify agronomic impacts while also measuring and assessing impacts on climate variables and the SDGs. Further structured evaluations, both within cropping regions and between regions within and between countries, would help to clarify the specific impacts and general application of impacts. This may require greater collaboration between economists, plant and soil scientists, climatologists and other social scientists, as measurement and modeling of each dimension is quite specific to the differing disciplines.

The science of genetic modification has confirmed that from a technology perspective, innovative products are capable of making important sustainability contributions. What would it mean if the barrier to further sustainability gains and climate change mitigation is now regulatory? At one level, it will draw attention to governments’ international commitments to improve sustainability, and the focus will shift to evaluating regulatory systems’ ability to respond to evidence of technologies that do contribute to sustainability. In turn, countries with product-based regulatory systems will continue to attract greater levels of not only R&D investments but will experience brain-gain by attracting scientists to work on innovative crop technologies. The portent is that process-based regulatory countries will experience a double loss in terms of both financial and human capital, and the global response to climate change using available technologies will be a patchwork. Foreseeably, the problem will worsen as innovative gene-edited crop varieties are commercialized raising again the question of who will benefit in the future as some governments, but not all, seek to reduce GHG emissions, increase carbon sequestration, improve food security, and mitigate climate change.

## References

[1] Khush GS. Genetically modified crops: the fastest adopted crop technology in the history of modern agriculture. Agric Food Secur. 2012;1(1):1–2. doi:10.1186/2048-7010-1-14.

[2] Brookes GBP, Barfoot P. Environmental impacts of genetically modified (GM) crop use 1996–2018: impacts on pesticide use and carbon emissions. GM Crops & Food. 2020;11(4):215–41. doi:10.1080/21645698.2020.1773198. 32706316 PMC7518756

[3] International Service for the Acquisition of Agri-biotech Applications (ISAAA). Biotech crops drive socio-economic development and sustainable environment in the new frontier. ISAAA Brief 55. 2020. https://www.isaaa.org/resources/publications/briefs/55/executivesummary/default.asp.

[4] Klümper W, Qaim M, Albertini E. A meta-analysis of the impacts of genetically modified crops. PLoS One. 2014;9(11):e111629. doi:10.1371/journal.pone.0111629. 25365303 PMC4218791

[5] Gleim S, Smyth SJ. Scientific underpinnings of biotechnology regulatory frameworks. N Biotechnol. 2018;42:26–32. doi:10.1016/j.nbt.2018.01.004. 29355665

[6] Hansen CW, Wingender AM. National and global impacts of genetically modified crops. Am Econ Revi Insights. 2023;5(2):224–40. doi:10.1257/aeri.20220144.

[7] Qaim M. Agricultural biotechnology in India: impacts and controversies. Chapter 9 In: Smyth SJ, Phillips PWB Castle D. editors. Handbook on agriculture, biotechnology and development. Cheltenham, UK:Edward Elgar Publishing Ltd; 2014:pp. 126–37.

[8] Tilman D. Global environmental impacts of agricultural expansion: the need for sustainable and efficient practices. Proc Natl Acad Sci. 1999;96(11):5995–6000. doi:10.1073/pnas.96.11.5995. 10339530 PMC34218

[9] Organisation for Economic Cooperation and Development. Making better policies for food systems. 2021. doi:10.1787/ddfba4de-en.

[10] Barrows G, Sexton S, Zilberman D. The impact of agricultural biotechnology on supply and land-use. Environ Dev Econ. 2014;19(6):676–703. doi:10.1017/S1355770X14000400.

[11] Mahaffey H, Taheripour F, Tyner WE. Evaluating the economic and environmental impacts of a global GMO ban. J Environ Prot. 2016;7(11):1522–1546.

[12] Taheripour F, Mahaffey H, Tyner WE. Evaluation of economic, land use, and land use emission impacts of substituting non-GMO crops for GMO in the US (no. 330-2016-13790). 2015.

[13] Zhang C, Wohlhueter R, Zhang H. Genetically modified foods: a critical review of their promise and problems. Food Sci Hum Wellness. 2016;5(3):116–23. doi:10.1016/j.fshw.2016.04.002.

[14] Brookes G, Barfoot P. Economic impact of GM crops. GM Crops Food Biotechnol Agric Food Chain. 2014;5(1):65–75. doi:10.4161/gmcr.28098. PMC5033197 24637520

[15] Brookes G. Farm income and production impacts from the use of genetically modified (GM) crop technology 1996-2020. GM Crops & Food. 2022;13(1):171–95. doi:10.1080/21645698.2022.2105626. 35983931 PMC9397136

[16] Phalan B, Onial M, Balmford A, Green RE. Reconciling food production and biodiversity conservation: land sharing and land sparing compared. Science. 2011;333(6047):1289–91. doi:10.1126/science.1208742. 21885781

[17] Sutherland C, Gleim S, Smyth SJ. Correlating genetically modified crops, glyphosate use and increased carbon sequestration. Sustainability. 2021;13(21):11679. doi:10.3390/su132111679.

[18] Awada L, Nagy C, Phillips PW, Ali G. Contribution of land use practices to GHGs in the Canadian Prairies crop sector. PLoS One. 2021;16(12):e0260946. doi:10.1371/journal.pone.0260946. 34919544 PMC8682883

[19] Kern M, Noleppa S, Schwarz G. Impacts of chemical crop protection applications on related CO^2^ emissions and CO^2^ assimilation of crops. Pest Manag Sci. 2012;68(11):1458–66. doi:10.1002/ps.3328. 22674852

[20] Canola Council of Canada (CCC). An agronomic and economic assessment of transgenic canola. 2001. http://www.canola-council.org/gmo_toc.aspx.

[21] Brimner TA, Gallivan GJ, Stephenson GR. Influence of herbicide‐resistant canola on the environmental impact of weed management. Pest Manage Sci Formerly Pesti Sci. 2005;61(1):47–52. doi:10.1002/ps.967. 15593073

[22] Kleter GA, Bhula R, Bodnaruk K, Carazo E, Felsot AS, Harris CA, Katayama A, Kuiper HA, Racke KD, Rubin B. et al. Altered pesticide use on transgenic crops and the associated general impact from an environmental perspective. Pest Manag Sci. 2007;63(11):1107–15. doi:10.1002/ps.1448. 17880042

[23] Brookes G, Barfoot P. Global impact of biotech crops: environmental effects, 1996–2008. AgBioForum. 2010;13:76–94.

[24] Leeson JY, Thomas AG, Beckie HJ, Brenzil CA, Hall LM, Andrews T, Brown KR, Van Acker RC. Herbicide-use trends in prairie canola production systems. 2006 soils and crops workshop [CD-ROM], extension division, Saskatoon, SK, Canada: University of Saskatchewan; March 2–3, 2006. p. 7.

[25] Smyth SJ, Gusta M, Belcher K, Phillips PW, Castle D. Changes in herbicide use after adoption of HR canola in Western Canada. Weed Technol. 2011;25(3):492–500. doi:10.1614/WT-D-10-00164.1.

[26] Perry ED, Ciliberto F, Hennessy DA, Moschini G. Genetically engineered crops and pesticide use in US maize and soybeans. Sci Adv. 2016a;2(8):e1600850. doi:10.1126/sciadv.1600850. 27652335 PMC5020710

[27] Dong F, Mitchell PD, Davis VM, Recker R. Impact of atrazine prohibition on the sustainability of weed management in Wisconsin maize production. Pest Manag Sci. 2017;73(2):425–34. doi:10.1002/ps.4298. 27101520

[28] Qaim M. Bt cotton in India: field trial results and economic projections. World Dev. 2003;31(12):2115–27. doi:10.1016/j.worlddev.2003.04.005.

[29] Subramanian A, Qaim M. The impact of bt cotton on poor households in rural India. J Dev Stud. 2010;46(2):295–311. doi:10.1080/00220380903002954.

[30] Kouser S, Qaim M. Impact of bt cotton on pesticide poisoning in smallholder agriculture: a panel data analysis. Ecol Econ. 2011;70(11):2105–13. doi:10.1016/j.ecolecon.2011.06.008.

[31] Pray C, Huang J. The impact of bt cotton in China. In: Kalaitzandonakes N, editor. The economic and environmental impacts of agbiotech. Boston, MA: Springer; 2003. pp. 223–42.

[32] Huang J, Mi J, Lin H, Wang Z, Chen R, Hu R, Rozelle S, Pray C. A decade of bt cotton in Chinese fields: assessing the direct effects and indirect externalities of Bt cotton adoption in China. Sci China Life Sci. 2010;53(8):981–91. doi:10.1007/s11427-010-4036-y. 20821297

[33] Ahmed AU, Hoddinott J, Abedin N, Hossain N. The impacts of GM foods: results from a randomized controlled trial of Bt eggplant in Bangladesh. Am J Agric Econ. 2021;103(4):1186–206. doi:10.1111/ajae.12162.

[34] Macall DM, Trabanino CR, Soto AH, Smyth SJ. Genetically modified maize impacts in Honduras: production and social issues. Transgenic Res. 2020;29(5):575–86. doi:10.1007/s11248-020-00221-y. 33175304

[35] Brookes G, Taheripour F, Tyner WE. The contribution of glyphosate to agriculture and potential impact of restrictions on use at the global level. GM Crops & Food. 2017;8(4):216–28. doi:10.1080/21645698.2017.1390637. 29035143 PMC5790413

[36] Walsh MJ, Harrington RB, Powles SB. Harrington seed destructor: a new nonchemical weed control tool for global grain crops. Crop Sci. 2012;52(3):1343–47. doi:10.2135/cropsci2011.11.0608.

[37] Statistic Canada. Table 32-10-0359-01, estimated areas, yield, production, average farm price and total farm value of principal field crops, in metric and imperial units. Ottawa, Canada: Queen’s Printer; 2022.

[38] Smyth SJ, Awada L). Assessment of Saskatchewan agricultural greenhouse gas emissions: sources, sinks and measures. Report submitted to the Global Institute for Food Security. 2018

[39] Grant B, Smith WN, Desjardins R, Lemke R, Li C. Estimated N^2^O and CO^2^ emissions as influenced by agricultural practices in Canada. Clim Change. 2004;65(3):315–32. doi:10.1023/B:CLIM.0000038226.60317.35.

[40] Shrestha BM, Desjardins RL, McConkey BG, Worth DE, Dyer JA, Cerkowniak DD. Change in carbon footprint of canola production in the Canadian prairies from 1986 to 2006. Renewable Energy. 2014;63:634–41. doi:10.1016/j.renene.2013.10.022.

[41] MacWilliam S, Sanscartier D, Lemke R, Wismer M, Baron V. Environmental benefits of canola production in 2010 compared to 1990: a life cycle perspective. Agric Syst. 2016;145:106–15.

[42] Biden S, Smyth SJ, Hudson D. The economic and environmental cost of delayed GM crop adoption: the case of Australia’s GM canola moratorium. GM Crops & Food. 2018;9(1):13–20. doi:10.1080/21645698.2018.1429876. 29359993 PMC5927647

[43] Kovak E, Blaustein-Rejto D, Qaim M. Genetically modified crops support climate change mitigation. Trends in Plant Science. 2022;27(7):627–629. doi:10.1016/j.tplants.2022.01.004. 35148945

[44] Hudson D, Richards R. Evaluation of the agronomic, environmental, economic, and coexistence impacts following the introduction of GM canola to Australia (2008-2010). AgBioforum. 2014;17:1–12.

[45] National Research Council. Environmental impacts of genetically engineered crops at the farm level. In: The impact of genetically engineered crops on farm sustainability in the United States. National Academies Press; 2010. p. 59–134.

[46] Fernandez-Cornejo J. First decade of genetically engineered crops in the United States. Darby, PA: DIANE Publishing; 2009.

[47] Givens WA, Shaw DR, Kruger GR, Johnson WG, Weller SC, Young BG, Wilson RG, Owen MDK, Jordan D. Survey of tillage trends following the adoption of glyphosate-resistant crops. Weed Technol. 2009;23(1):150–55. doi:10.1614/WT-08-038.1.

[48] Harrington J, Byrne PF, Frank B, Nissen SJ, Westra P, Ellsworth PC, Henry WB. Perceived consequences of herbicide-tolerant and insect-resistant crops on integrated pest management strategies in the Western United States: results of an online survey. AgBioforum. 2009;12:412–21.

[49] Fernandez-Cornejo J, Hallahan C, Nehring RF, Wechsler S, Grube A. Conservation tillage, herbicide use, and genetically engineered crops in the United States: the case of soybeans. AgBioforum. 2013;15:231–41.

[50] Fernandez-Cornejo J, Klotz-Ingram C, Jans S. Farm-level effects of adopting herbicide-tolerant soybeans in the USA. J Agric Appl Econ. 2002;34(1):149–63. doi:10.1017/S1074070800002200.

[51] Perry ED, Moschini G, Hennessy DA. Testing for complementarity: glyphosate tolerant soybeans and conservation tillage. Am J Agric Econ. 2016b;98(3):765–84. doi:10.1093/ajae/aaw001.

[52] Molberg ES, McCurdy EV, Wenhardt A, Dew DA, Dryden RD. Minimum tillage requirements for summerfallow in western Canada. Can J Soil Sci. 1967;47(3):211–16. doi:10.4141/cjss67-033.

[53] Carlyle WJ. The decline of summerfallow on the Canadian Prairies. Can Geographer. 1997;41(3):267–80. doi:10.1111/j.1541-0064.1997.tb01313.x.

[54] Boehm M, Junkins B, Desjardins R, Kulshreshtha S, Lindwall W. Sink potential of Canadian agricultural soils. Clim Change. 2004;65(3):297–314. doi:10.1023/B:CLIM.0000038205.09327.51.

[55] Mikha MM, Benjamin JG, Vigil MF, Nielson DC. Cropping intensity impacts on soil aggregation and carbon sequestration in the central great plains. Soil Sci Soc Am J. 2010;74(5):1712–19. doi:10.2136/sssaj2009.0335.

[56] Ogle SM, Breidt FJ, Paustian K. Agricultural management impacts on soil organic carbon storage under moist and dry climatic conditions of temperate and tropical regions. Biogeochemistry. 2005;72(1):87–121. doi:10.1007/s10533-004-0360-2.

[57] Sperow M. Estimating carbon sequestration potential on US agricultural topsoils. Soil Tillage Res. 2016;155:390–400. doi:10.1016/j.still.2015.09.006.

[58] Rosenzweig ST, Schipanski ME. Landscape-scale cropping changes in the high plains: economic and environmental implications. Environ Res Lett. 2019;14(12):124088. doi:10.1088/1748-9326/ab5e8b.

[59] Campbell CA, Zentner RP, Gameda S, Blomert B, Wall DD. Production of annual crops on the Canadian prairies: trends during 1976–1998. Can J Soil Sci. 2002;82(1):45–57. doi:10.4141/S01-046.

[60] Hall SJ, Russell AE, Moore AR. Do corn-soybean rotations enhance decomposition of soil organic matter? Plant Soil. 2019;444(1):427–42. doi:10.1007/s11104-019-04292-7.

[61] Gan YT, Campbell CA, Janzen HH, Lemke RL, Basnyat P, McDonald CL. Carbon input to soil from oilseed and pulse crops on the Canadian prairies. Agr Ecosyst Environ. 2009;132(3–4):290–97. doi:10.1016/j.agee.2009.04.014.

[62] Bakker MM, Govers G, Jones RA, Rounsevell MD. The effect of soil erosion on Europe’s crop yields. Ecosystems. 2007;10(7):1209–19. doi:10.1007/s10021-007-9090-3.

[63] Jarecki MK, Lal R. Crop management for soil carbon sequestration. CRC Crit Rev Plant Sci. 2003;22(6):471–502. doi:10.1080/713608318.

[64] Sauer TJ, Hatfield JL, Prueger JH. Corn residue age and placement effects on evaporation and soil thermal regime. Soil Sci Soc Am J. 1996;60(5):1558–64. doi:10.2136/sssaj1996.03615995006000050039x.

[65] Carpenter JE. Impact of GM crops on biodiversity. GM Crops. 2011;2(1):7–23. doi:10.4161/gmcr.2.1.15086. 21844695

[66] McConkey B, Luce MS, Grant B, Smith W, Padbury G, Brandt K, Cerkowniak D. Saskatchewan soil conservation association prairie soil carbon balance project: monitoring SOC change across saskatchewan farms from 1996 to 2018. Change In SOC At Field Level Compon. February 2020. https://static1.squarespace.com/static/5fc882025388527f26b77665/t/5ff2b6fa0db4f45ccbebd302/1609742076069/2020-0223+PSCB+Report+2020+Final.pdf.

[67] Olson KR, Al-Kaisi M, Lal R, Morton LW. Soil ecosystem services and intensified cropping systems. J Soil Water Conserv. 2017;72(3):64A–9A. doi:10.2489/jswc.72.3.64A.

[68] Lal R. Soil carbon sequestration impacts on global climate change and food security. Science. 2004;304(5677):1623–27. doi:10.1126/science.1097396. 15192216

[69] Powlson DS, Whitmore AP, Goulding KW. Soil carbon sequestration to mitigate climate change: a critical re‐examination to identify the true and the false. Eur J Soil Sci. 2011;62(1):42–55. doi:10.1111/j.1365-2389.2010.01342.x.

[70] Campbell CA, Zentner RP, Selles F, Liang BC, Blomert B. Evaluation of a simple model to describe carbon accumulation in a brown chernozem under varying fallow frequency. Can J Soil Sci. 2001;81(4):383–94. doi:10.4141/S00-082.

[71] West TO, Post WM. Soil organic carbon sequestration rates by tillage and crop rotation: a global data analysis. Soil Sci Soc Am J. 2002;66(6):1930–46. doi:10.2136/sssaj2002.1930.

[72] Nemo, Klumpp K, Coleman K, Dondini M, Goulding K, Hastings A, Jones M, Leifeld J, Osborne B, Saunders M, Scott T. Soil organic carbon (SOC) equilibrium and model initialisation methods: an application to the Rothamsted carbon (RothC) model. Environ Model Assess. 2017;22(3):215–29. doi:10.1007/s10666-016-9536-0.

[73] Wutzler T, Reichstein M. Soils apart from equilibrium–consequences for soil carbon balance modelling. Biogeosciences. 2007;4(1):125–36. doi:10.5194/bg-4-125-2007.

[74] Nath AJ, Rattan LAL. Effects of tillage practices and land use management on soil aggregates and soil organic carbon in the north Appalachian region, USA. Pedosphere. 2017;27(1):172–76. doi:10.1016/S1002-0160(17)60301-1.

[75] Paustian K. Modelling soil organic matter dynamics - global challenges. In: Rees RM, Ball BC, Campbell CD Watson CA. editors Sustainable management of soil organic matter. Oxon, UK: CABI Press; 2000. p. 45–53.

[76] McConkey BG, Liang BC, Campbell CA, Curtin D, Moulin A, Brandt SA, Lafond GP. Crop rotation and tillage impact on carbon sequestration in Canadian prairie soils. Soil Tillage Res. 2003;74(1):81–90. doi:10.1016/S0167-1987(03)00121-1.

[77] Liebig MA, Morgan JA, Reeder JD, Ellert BH, Gollany HT, Schuman GE. Greenhouse gas contributions and mitigation potential of agricultural practices in northwestern USA and western Canada. Soil Tillage Res. 2005;83(1):25–52. doi:10.1016/j.still.2005.02.008.

[78] Aziz I, Mahmood T, Islam KR. Effect of long term no-till and conventional tillage practices on soil quality. Soil Tillage Res. 2013;131:28–35. doi:10.1016/j.still.2013.03.002.

[79] Angers DA, Eriksen-Hamel NS. Full‐inversion tillage and organic carbon distribution in soil profiles: a meta‐analysis. Soil Sci Soc Am J. 2008;72(5):1370–74. doi:10.2136/sssaj2007.0342.

[80] Blanco-Canqui H, Lal R. No‐tillage and soil‐profile carbon sequestration: An on‐farm assessment. Soil Sci Soc Am J. 2008;72(3):693–701. doi:10.2136/sssaj2007.0233.

[81] VandenBygaart AJ, Bremer E, McConkey BG, Ellert BH, Janzen HH, Angers DA, Carter MR, Drury CF, Lafond GP, McKenzie RH. Impact of sampling depth on differences in soil carbon stocks in long‐term agroecosystem experiments. Soil Sci Soc Am J. 2011;75(1):226–34. doi:10.2136/sssaj2010.0099.

[82] Yanni S, Rajsic P, Wagner-Riddle C, Weersink A. 2018). A review of the efficacy and cost-effectiveness of on-farm BMPs for mitigating soil-related GHG emissions. Working Paper Series–WP 18-05. Institute for the Advanced Study of Food and Agricultural Policy. Department of Food, Agriculture, and Resource Economics. University of Guelph. http://ageconsearch.umn.edu/record/276270/files/SynthesisofGH.

[83] Lee S, Clay DE, Clay SA. Impact of herbicide tolerant crops on soil health and sustainable agriculture crop production. In: Songstad D, Hatfield J, and Tomes D. editors. Convergence of food security, energy security and sustainable agriculture. Biotechnology in agriculture and forestry. Vol. 67. Berlin, Heidelberg:Springer; 2014. p. 211–236.

[84] Fiodor A, Singh S, Pranaw K. The contrivance of plant growth promoting microbes to mitigate climate change impact in agriculture. Microorganisms. 2021;9(9):1841. doi:10.3390/microorganisms9091841. 34576736 PMC8472176

[85] Radcliffe J. 2019). Reducing crime: a companion for police leaders. http://reducingcrime.com.

[86] Entine J, Felipe MSS, Groenewald J-H, Kershen DL, Lema M, McHughen A, Nepomuceno AL, Ohsawa R, Ordonio RL, Parrott WA. et al. Regulatory approaches for genome edited agricultural plants in select countries and jurisdictions around the world. Transgenic Res. 2021;3(4):551–84. doi:10.1007/s11248-021-00257-8. PMC8316157 33970411

[87] Smyth SJ. Contributions of genome editing technologies towards improved nutrition, environmental sustainability and poverty reduction. Front Genome Ed. 2022;4:1–9. doi:10.3389/fgeed.2022.863193. PMC8968197 35373188

[88] Smyth SJ, Lassoued R. Agriculture R&D implications of the CJEU’s gene-editing mutagenesis ruling. Trends Biotechnol. 2018;37(4):337–40. doi:10.1016/j.tibtech.2018.09.004. 30293646

[89] Smyth SJ, McDonald J, Falck-Zepeda J. Investment, regulation, and uncertainty: managing new plant breeding techniques. GM Crops & Food. 2014;5(1):1–14. doi:10.4161/gmcr.27465. 24499745 PMC5033172

[90] Wesseler J. The EU’s farm-to-fork strategy: an assessment from the perspective of agricultural economics. Appl Econ Perspect Policy. 2022;44(4):1826–43. doi:10.1002/aepp.13239.

[91] Food and Agriculture Organization. The 10 elements of agroecology: guiding the transition to sustainable food and agricultural systems. 2020. https://www.fao.org/3/i9037en/i9037en.pdf.

